# Cognitive impact of COVID-19: looking beyond the short term

**DOI:** 10.1186/s13195-020-00744-w

**Published:** 2020-12-30

**Authors:** Scott Miners, Patrick G. Kehoe, Seth Love

**Affiliations:** Dementia Research Group, Bristol Medical School (THS), University of Bristol, Learning & Research level 1, Southmead Hospital, Bristol, BS10 5NB UK

**Keywords:** COVID-19, SARS-CoV-2, Stroke, White matter ischaemia, Angiotensin-converting enzyme-2, Angiotensin-converting enzyme inhibitors, Angiotensin receptor blockers, Cognitive impairment, Dementia

## Abstract

COVID-19 is primarily a respiratory disease but up to two thirds of hospitalised patients show evidence of central nervous system (CNS) damage, predominantly ischaemic, in some cases haemorrhagic and occasionally encephalitic. It is unclear how much of the ischaemic damage is mediated by direct or inflammatory effects of virus on the CNS vasculature and how much is secondary to extracranial cardiorespiratory disease. Limited data suggest that the causative SARS-CoV-2 virus may enter the CNS via the nasal mucosa and olfactory fibres, or by haematogenous spread, and is capable of infecting endothelial cells, pericytes and probably neurons. Extracranially, SARS-CoV-2 targets endothelial cells and pericytes, causing endothelial cell dysfunction, vascular leakage and immune activation, sometimes leading to disseminated intravascular coagulation. It remains to be confirmed whether endothelial cells and pericytes in the cerebral vasculature are similarly targeted. Several aspects of COVID-19 are likely to impact on cognition. Cerebral white matter is particularly vulnerable to ischaemic damage in COVID-19 and is also critically important for cognitive function. There is accumulating evidence that cerebral hypoperfusion accelerates amyloid-β (Aβ) accumulation and is linked to tau and TDP-43 pathology, and by inducing phosphorylation of α-synuclein at serine-129, ischaemia may also increase the risk of development of Lewy body disease. Current therapies for COVID-19 are understandably focused on supporting respiratory function, preventing thrombosis and reducing immune activation. Since angiotensin-converting enzyme (ACE)-2 is a receptor for SARS-CoV-2, and ACE inhibitors and angiotensin receptor blockers are predicted to increase ACE-2 expression, it was initially feared that their use might exacerbate COVID-19. Recent meta-analyses have instead suggested that these medications are protective. This is perhaps because SARS-CoV-2 entry may deplete ACE-2, tipping the balance towards angiotensin II-ACE-1-mediated classical RAS activation: exacerbating hypoperfusion and promoting inflammation. It may be relevant that *APOE* ε4 individuals, who seem to be at increased risk of COVID-19, also have lowest ACE-2 activity. COVID-19 is likely to leave an unexpected legacy of long-term neurological complications in a significant number of survivors. Cognitive follow-up of COVID-19 patients will be important, especially in patients who develop cerebrovascular and neurological complications during the acute illness.

## Background

COVID-19, caused by severe acute respiratory syndrome coronavirus-2 (SARS-CoV-2), is primarily a respiratory disease but has the capacity to damage other organs including the brain. Similarly to the severe acute respiratory syndrome (SARS) and Middle East respiratory syndrome (MER) viruses [1–3], SARS-CoV-2 targets the brain (reviewed [4]) and a growing number of case reports and cohort studies indicate significant neurological disturbance in COVID-19 patients (reviewed [5]). Central nervous system (CNS) involvement including non-specific encephalopathy (headache, confusion, and disorientation) was first documented in 53/214 (25%) hospitalised patients in Wuhan, China [6]. More recent studies in Europe have reported higher rates of CNS involvement: 69% of 58 hospitalised patients in a French Study [7], and 31% of 125 cases with altered mental state, including psychosis and neurocognitive changes, in a recent UK survey [8]. A recent report described a ‘dysexecutive syndrome consisting of inattention, disorientation, or poorly organised movements in response to command’ in 33% of 43 patients discharged from hospital [7]. Moreover, neuroradiological evidence of microstructural damage and disruption of functional brain integrity at 3-month follow-up in recovered COVID-19 patients [9] indicates potential long-term neurological consequences in severely affected COVID-19 patients (reviewed [10]). Acute cerebrovascular disease (CVD), typically presenting as ischaemic stroke but occasionally as intracerebral haemorrhage (ICH), has emerged as an important clinical feature in COVID-19 (reviewed in [5]). There have also been multiple case reports of encephalitis with brain-stem involvement (reviewed in [5]). CNS involvement with neurological presentation is more frequent in older and more severely ill COVID-19 patients [6]. Based on the minimum prevalence of neurological complications in SARS and MERS, Ellul et al. [5] estimated that of the reported 4.8 million COVID-19 cases at the time, 1805–9671 had developed CNS complications.

Human coronaviruses are known to target the CNS and cause damage by direct neurotoxicity or activation of the host immune response [1]. The propensity of SARS-CoV-2 to cause cerebral vascular injury greatly increases the risk of chronic brain damage, not only because of the cumulative destructive effect of multifocal cerebral ischaemia or haemorrhage, but potentially also through chronic post-infective complications of CVD, including endothelial and blood-brain barrier (BBB) dysfunction and upregulation of pro-inflammatory cytokines within the brain [11]. Long-term cognitive decline and neurodegeneration, with associated hippocampal atrophy [12], were previously reported to complicate systemic inflammation associated with severe sepsis [13, 14]. Acute respiratory distress syndrome (ARDS), a common clinical presentation in COVID-19 patients, is also associated with cognitive decline and neurodegeneration [15, 16]. Long-term follow-up of COVID-19 patients that includes detailed cognitive assessment will be important, to determine the extent and prevalence of long-term neurological and psychiatric consequences of COVID-19 [17], especially in patients who develop cerebrovascular and neurological complications during the acute illness.

In this review, we discuss the pathophysiological processes and risk factors shared by COVID-19 and dementia, focussing particularly on the role of cerebrovascular disease and the involvement of the renin-angiotensin system (RAS) (Table 1). We consider whether SARS-CoV-2 infection may increase the risk of later developing dementia, particularly in people with underlying cerebrovascular disease and high-risk co-morbidities, such as diabetes and hypertension.

**Table 1:** Pathophysiological processes contributing to increased risk of chronic neurological disease, including dementia, in COVID-19 patients

	References
1. Hypoxia and cerebral hypoperfusion secondary to cardiorespiratory disease	[25, 26]
- Hypoxic-ischaemic brain injury, diffuse white matter damage	
2. Coagulopathy, with thrombotic occlusion of cerebral blood vessels	[22]
- Cerebral artery thrombosis, disseminated intravascular coagulation	
3. Cerebral microvascular damage and dysfunction	[23, 24]
- Endotheliitis, pericyte damage, BBB leakiness, neurovascular dysfunction, impaired autoregulation, impaired vascular/para-vascular drainage	
4. Dysregulation of renin-angiotensin system	[125–127, 160, 161]
- Loss of regulatory RAS and overactivity of classical RAS signalling	
5. SARS-CoV-2 encephalitis / post-infective encephalitis (rare)	[27, 28, 38], reviewed in [5]
- CNS viral neuroinvasion via olfactory nerve fibres or vasculature/post-infective immune injury to CNS	

### Cerebral vascular disease (CVD) is common in severe COVID-19

Unlike in SARS and MERS, COVID-19 patients are at substantial risk of developing acute CVD. Studies to date indicate that CVD has affected 2–6% of hospitalised patients with COVID-19 (reviewed in [5]). In a Spanish cohort, 23 of 1683 patients (1.4%) developed CVD, with cerebral ischaemia accounting for 74% and ICH for 23% of the 23 cases [18]. Amongst COVID-19 patients with neurological complications, the reported incidence of CVD is much higher. Acute CVD was diagnosed in 77% of 56 patients admitted to a neurology ward in Italy [19]. In a recent UK-wide survey of 153 COVID-19 cases with neurological and/or psychiatric disturbance, most patients (62% of 125), for whom a full clinical dataset was available, had had a cerebrovascular event, compared to 31% with encephalopathy; of those with CVD, 74% had presented with ischaemic stroke, 12% with ICH, and 1% with CNS vasculitis [8]. A common theme across most of these studies is the predominance of CVD in older patients in association with more severe disease, and in those with co-morbidities, including hypertension, diabetes and underlying cerebrovascular disease [20]. However, large-vessel stroke has also been reported in younger adults with COVID-19 [21].

**Fig. 1:**
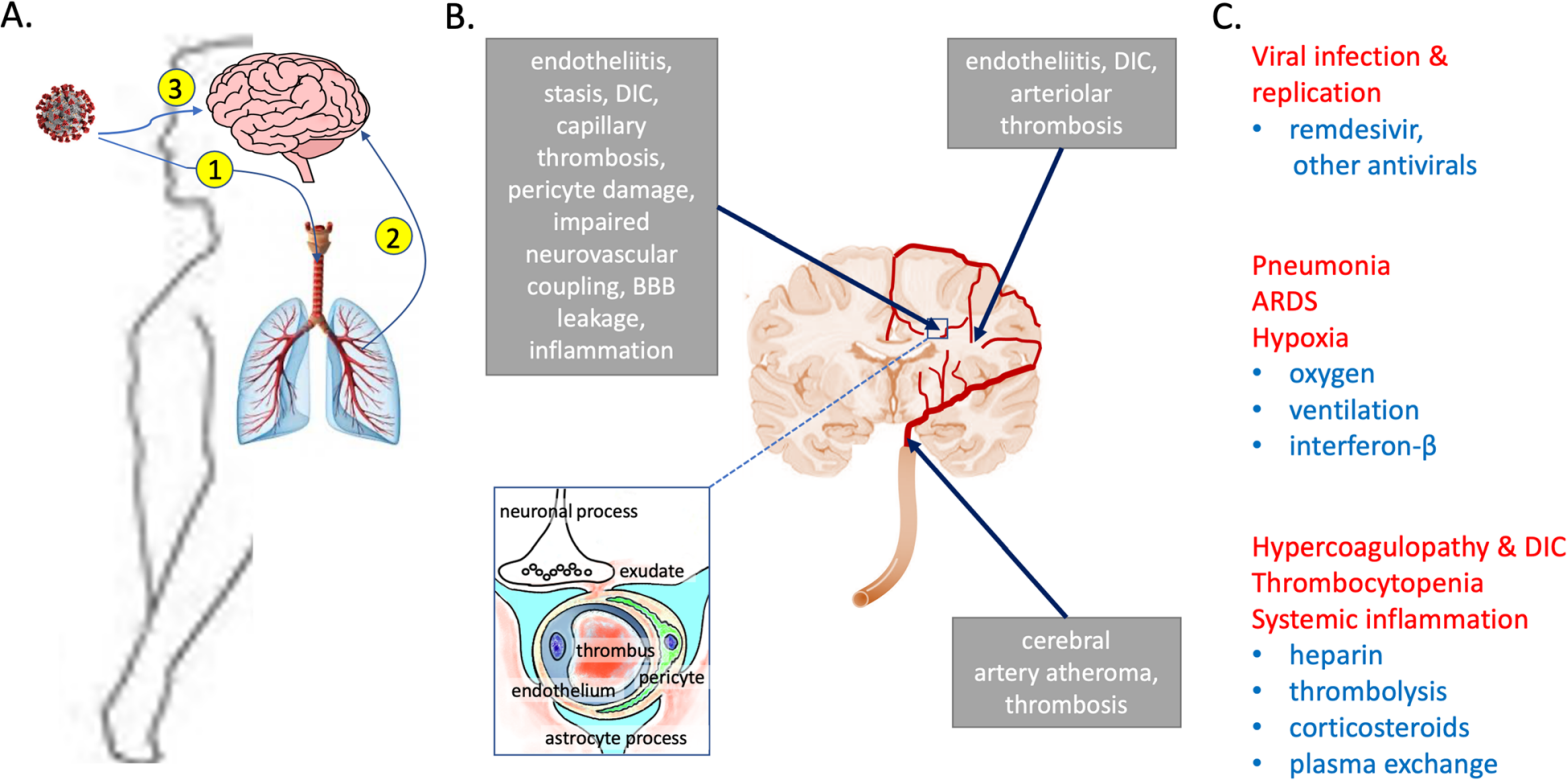
Mechanisms of cerebrovascular damage in COVID-19. **a** The virus reaches the central nervous system through inhalation (1) and lung infection followed by haematogenous spread (2), or through the nasal mucosa and olfactory nerve fibres (3). **b** A high proportion of COVID-19 patients with severe disease develop cerebrovascular disease. In addition to hypoxic-ischaemic brain damage from compromised respiratory and cardiovascular function, the virus may cause large-vessel stroke, multiple small infarcts and foci of haemorrhage, and diffuse ischaemic white matter damage and oedema. Putative mechanisms are illustrated in the diagram. **c** Already, considerable progress has been made in preventing or ameliorating cerebrovascular damage in COVID-19. Some of the approaches are listed here

The pathophysiology of CVD in COVID-19 has yet to be fully determined (Fig. 1). Inflammation-induced disseminated intravascular coagulation (DIC), often complicated by pulmonary embolism, has been documented in a high proportion of patients with neurovascular complications and is likely to be a major contributor to most acute CVD events in COVID-19 [22], especially in younger healthy adults. A recent review [23] highlighted a number of pathways involving localised endothelial cell dysfunction, vascular leakage and unregulated immune activation that contribute to DIC formation in ARDS in COVID-19 patients. Activation of the kallikrein-bradykinin system leading to reduced blood flow, upregulation of adhesion molecules that mediate leucocyte recruitment, activation of platelets and neutrophils, and increased inflammation and immune surveillance all potentially contribute to vascular damage (and pulmonary injury) in COVID-19 patients. SARS-CoV-2 has also been shown to target and infect endothelial cells in vascular beds in multiple tissues [24], but it remains to be confirmed whether endothelial cells in the cerebral vasculature are similarly targeted.

Most studies to date have implicated vascular dysfunction and ischaemic damage in the major neurological complications associated with COVID-19. Post-mortem neuropathological examination in 18 COVID-19 cases showed all to have acute hypoxic-ischaemic brain injury affecting the cerebrum and cerebellum, with rare foci of perivascular inflammation in two of the brains but no convincing evidence of virus within the CNS [25]. MR imaging of autopsy brains from deceased COVID-19 patients within 24 h of death revealed white matter changes including foci of haemorrhage in two cases and evidence of posterior reversible encephalopathy syndrome in another [26]. Plasma markers of neuronal and astrocyte injury (neurofilament light chain protein and glial fibrillary acidic protein) were elevated in COVID-19 patients and associated with disease severity [27]. The authors concluded that further studies were needed to assess the relationship of brain damage to ischaemic and inflammatory processes. The key unanswered question is how much damage is mediated by direct effects of virus on the CNS parenchyma or vasculature (damage that would be expected to persist after the virus is cleared from the CNS), how much is indirect CNS vascular injury mediated by immune activation, and how much is hypoxic-ischaemic damage secondary to the extracranial effects of the virus on the respiratory and cardiovascular systems.

### SARS-CoV-2 infects human brain

Both SARS-CoV-2 antigen and RNA have been detected within brain tissue in human post-mortem studies, the antigen mainly within the medulla and lower cranial nerves [28]. SARS-CoV-2 was detected in cerebrospinal fluid from a patient with viral encephalitis [29] and was observed at autopsy in neural and capillary endothelial cells in COVID-19 brain tissue [30]. These observations need to be confirmed in further studies, particularly given the high Ct values used for the PCR detection of viral RNA and the difficulty of electron microscopic interpretation of virus-like particles. Although retrograde axonal transport via the olfactory bulb, associated with anosmia, is a potential route for neuroinvasion, it is likely that the cerebral vasculature plays a more important role in entry of the virus into the CNS. ACE-2, the principal SARS-CoV-2 receptor, is highly expressed by endothelial cells [31] and pericytes throughout the body [32] and analysis of publicly available databases indicates that ACE-2 is also expressed in the brain [33].

Despite apparent low levels of ACE-2 mRNA within the brain [34–37], SARS-Cov-2 infects induced pluripotent stem cell-derived human neural stem and progenitor cells, neurospheres, and cortical neurons with brain organoids (all of which express ACE-2) [38–41]. These data suggest that the mRNA levels do not necessary reflect ACE-2 protein or enzyme activity within the brain, although we would point out that some of the information (e.g. [38–41]) has been published only on preprint servers; peer review may lead to modification of the conclusions. We and others have detected ACE-2 immunohistochemically within the cerebral vasculature in human post-mortem brains [38, 42] and a pre-published study by the Betsholtz lab indicates that ACE-2 is also enriched in brain pericytes [43]. In addition to ACE-2, other docking receptors for SARS-CoV-2 have been identified, including basigin (BSG, CD147) (preprint [44]) and neuropilin (NRP1) (preprint [45]), and these are highly expressed in endothelial cells and pericytes [46]. These receptors may have an important role to play, alongside or separately to ACE-2, in viral entry and disease pathogenesis (reviewed [46]).

Activation of the cerebral endothelium in a range of disease states, including AD, is associated with increased expression of integrins and selectins that are responsible for the attachment, tethering and passage of immune cells across the BBB. This leads to infiltration of brain tissue by immune cells, including neutrophils, monocytes, and lymphocytes, contributing to the pathogenesis of disease (reviewed [47]). In view of the activation of endothelium and inflammatory cell infiltration in lung and other tissues in COVID-19, it is conceivable that activation of the cerebral endothelium and infiltration by immune cells also contributes to neurological disturbance in many patients, although this too remains to be determined.

Pericytes are mural cells located within the basement membrane of microvessels [48] and communicate with endothelial cells to maintain integrity of the BBB [49] and regulate essential vascular functions: blood flow [50] and neurovascular coupling, endothelial transcytosis [51], and angiogenesis [52]. Transcriptomic analysis of murine heart [32, 53] and brain [54–56] indicates that pericytes express high levels of ACE-2 and are therefore likely targets of SARS-CoV-2. Lung biopsies from 4 hospitalised Covid-19 patients revealed a dramatic reduction in pericyte coverage of alveolar capillaries in addition to thickening of the capillary wall [57]. Pericyte degeneration and the consequent disruption of endothelial signalling and homeostasis are likely to be important contributors to vascular instability in COVID-19 [32]. In pericyte-deficient mice (Pdgfrb^ret/ret^), levels of von Willebrand factor, which promotes platelet aggregation and coagulation, are elevated, suggesting that pericyte loss contributes to the pro-angiogenic response in COVID-19 patients [43]. These studies implicate pericyte dysfunction as a mediator of pathophysiology in COVID-19. It is not yet known whether pericytes within the brain degenerate or become dysfunctional in COVID-19 patients with neurological manifestations.

A recent study has provided further evidence of the neuroinvasive potential of SARS-CoV-2 [38]. The authors demonstrated ACE-2-dependent infection of nerve cells within human brain organoids, and hypoxia-like metabolic changes and damage in neighbouring uninfected cells. Expression of humanised ACE-2 within the brain of mice experimentally infected with SARS-CoV-2 caused vascular remodelling throughout the cortex and greatly increased their mortality. The authors examined brain tissue from three COVID-19 patients and reported that SARS-CoV-2 spike protein could be detected immunohistochemically within the walls of small vessels cortical adjacent to microinfarcts. They also reported spike protein immunopositivity in some cortical neurons. However, these findings need to be confirmed.

### The types of cerebral ischaemic damage seen in COVID-19 are major contributors to cognitive decline and dementia

Pre-existing dementia ranks as one of the most significant risk factors, or co-morbidities, in COVID-19. Retrospective assessment of UK health records in the UK OpenSAFELY platform indicated a hazard ratio of 2.16 (fully adjusted model) in association with pre-existing dementia/stroke [58]. An odds ratio of 3.07 was associated with dementia in a UK biobank community study [59]. The reasons for the increased risk and mortality in patients with pre-existing dementia have been well reviewed [60]. What is perhaps not as widely appreciated is that the types of brain damage seen in COVID-19 are themselves major contributors to cognitive decline and dementia.

Ischaemic brain damage is the defining pathological process in vascular dementia (VaD), and stroke is a major risk factor for dementia [61, 62]. Thromboembolic occlusion of cerebral blood vessels, a major complication of DIC, can cause a wide spectrum of neurological deficits, including cognitive impairment or dementia. Single or multiple infarcts, as a result of thromboembolism affecting major cerebral arteries, are estimated to account for approximately 20% of dementia cases associated with stroke [63]. It is probable that acute large cerebral vascular occlusion associated with hypercoagulability in severely affected COVID-19 patients will increase the risk of dementia to some extent.

Small vessel disease (SVD) accounts for about 20% of all strokes [64] but about 80% of stroke-related dementia cases [63], and is the most common cause of vascular cognitive impairment. SVD-associated neuroimaging abnormalities of the white matter and arteriolosclerosis of cerebral microvessels can be demonstrated in about 50% of all patients with dementia [65, 66]. Co-morbidities of SVD include hypertension and diabetes [67] (both also risk factors for severe COVID-19). Hypercoagulability and disseminated intravascular coagulation that affect many patients with severe COVID-19 are likely to reduce perfusion through small intracerebral vessels more than through larger ones. SARS-COV-2 induces endothelial dysfunction [23] and infects vascular beds in multiple tissues [24]. Cerebral white matter is particularly vulnerable to changes in cerebral blood flow, as would be expected in association with diffuse small vessel dysfunction and has been reported in COVID-19 [68–70]. The integrity of subcortical white matter is critically important for maintenance of cognitive function [71, 72], and one of the consequences of white matter damage in COVID-19 is likely to be cognitive impairment. This was highlighted by the neuroradiological demonstration that damage to white matter and disruption of functional integrity in brain regions such as the hippocampus, at 3-month follow-up in recovered COVID-19 patients, was associated with memory loss [9].

The pathophysiology of SVD remains incompletely understood but damage to endothelial cells and pericytes, as well as BBB leakiness, are contributors to SVD-related brain injury (reviewed [66, 73]), which are likely to be exacerbated in severe COVID-19. Endothelial dysfunction and pericyte loss are associated with the cerebral influx and accumulation of toxic constituents of the plasma, such as fibrinogen, leading to oligodendrocyte damage and myelin loss [74]. Fibrinogen-mediated activation of the bone morphogenetic protein signalling pathway prevents maturation of oligodendrocyte precursor cells and restricts oligodendrocyte maturation and remyelination [75]. The infiltration of immune cells across a damaged BBB may also contribute to white matter damage and cognitive decline in dementia and perhaps COVID-19 (reviewed [47]). In addition, endothelial dysfunction and loss of pericytes are likely to impair the clearance of cerebral metabolites, including Aβ peptides, that are toxic when present in excess. Impaired drainage of metabolites including Aβ is implicated in the development of cerebral amyloid angiopathy and Alzheimer’s disease [76, 77] and ineffective drainage of solutes probably accounts for enlargement of perivascular spaces in patients with SVD (reviewed [78, 79] and cerebral amyloid angiopathy ([80–82]).

Several other factors may impact on cerebral perfusion during systemic infection. Increased blood viscosity tends to slow capillary transit and limit oxygen delivery. Damage to the glycocalyx, a carbohydrate-enriched matrix on the luminal side of capillaries may impair perfusion and exacerbate ischaemia [83, 84]. Many of the deleterious alterations to small vessels in systemic infection are exacerbated by the same risk factors that predispose to severe COVID-19, such as ageing, hypertension, diabetes, and obesity.

Post-mortem and neuroimaging studies indicate that ischaemic damage to the cerebral white matter is present in up to two thirds of patients with AD [85–87]. Although cerebral amyloid angiopathy may contribute to the damage, in most cases the ischaemia-related damage probably results from a combination of arteriolosclerotic SVD and non-structural vascular dysfunction (reviewed [88]). A series of recent neuroimaging studies has shown that ischaemic white matter damage occurs at a very early stage of AD, accelerates progression of the disease and contributes to the cognitive decline [89–93]. These clinical observations are supported by experimental studies showing that brain ischaemia accelerates Aβ accumulation, through a combination of dysregulated processing of amyloid-β precursor protein (APP) and impaired Aβ clearance (reviewed [87]) and that, in turn, Aβ peptides mediate vasoconstriction, by inducing contraction of pericytes [94] and vascular smooth muscle cells [95]. Studies of microvascular endothelial cell monolayers [96, 97], human APP transgenic mouse models [98], and on human post-mortem brain tissue [99] indicate that Aβ peptides also impair BBB function, in part by reducing expression of tight junction proteins. Pericyte degeneration in AD is associated with BBB breakdown [100–102]. Pericyte loss accelerates Aβ pathology and induces tau pathology and cognitive decline in human APP mice [103]. In human brain tissue from AD patients, a decline in the level of the pericyte marker, platelet-derived growth factor-β (PDGFRβ), was associated with increased Aβ level and reduced cerebral perfusion [104]. Aβ peptides are toxic to human brain pericytes in culture [105] and analysis of cerebrospinal fluid indicates that the level of soluble PDGFRβ, a marker of pericyte injury and BBB leakiness, is elevated in the elderly in association with the earliest detectable changes in cognitive performance [89, 90].

There is increasing evidence that cerebral hypoperfusion is also linked to tau pathology. In clinically normal adults with positron emission tomography (PET) evidence of cerebral Aβ accumulation, those who also had an increased cardiovascular disease risk score were significantly more likely to show evidence of tau accumulation as well [106]. In patients with mild cognitive impairment, increased cerebrovascular burden was associated with elevated PET-Tau signal and worse cognitive performance, independently of Aβ-PET [107]. Several experimental studies have shown that modelling of cerebral hypoperfusion increases tau phosphorylation: in adult Wistar rats [108], transgenic mice with Aβ and tau accumulation [109], and rat and human brain slices exposed to oxygen and glucose deprivation [110]. In a recent autopsy study, elevated levels of soluble tau and insoluble phospho-tau were associated with lower levels of endothelial tight junction proteins, claudin-5 and occludin, in AD [111]. Tau-overexpressing mice were shown to have abnormal blood vessel morphology and increased vessel density [112]. A recent study found impaired neurovascular coupling, prior to neurodegeneration, in young (2–3 months) mice expressing mutant tau [113]. There are therefore clinical and experimental data suggesting a bidirectional relationship between cerebral vascular dysfunction and pathological tau. There is evidence from recent studies that TDP-43 pathology, likewise, is associated with cerebral vascular dysfunction, including pericyte loss and small vessel disease [114, 115]. Lastly, cerebral ischaemia induces phosphorylation of α-synuclein at serine-129; this is the predominant disease-related modification of α-synuclein in Lewy bodies and neurites in Parkinson disease, and dementia with Lewy bodies, and also is significantly associated with concomitant AD pathology in these Lewy body diseases [116].

Both cerebral ischaemia and systemic inflammation can induce endothelial activation, with increased expression of integrins and selectins leading to adhesion and transendothelial migration of leukocytes into the brain parenchyma. Endothelial activation, with recruitment of leukocytes, has also been shown in AD [47, 117]. Leukocytes enter the brain through post-capillary venules within the parenchyma, and to a lesser extent, vessels in the leptomeninges and choroid plexus. Activated neutrophils in cerebral vessels and parenchyma were found to contribute to gliosis and cognitive impairment in human APP mice [118]. In APP/PS1 mice, respiratory infection increased infiltration of the brain by interferon γ- and interleukin-17-producing T cells and natural killer T cells, accompanied by gliosis and enhanced deposition of Aβ [119]. Monocytes are the most common type of peripheral immune cell that migrates via the BBB into the brain in AD. CCL2, the major ligand for CCR2 expressed on monocytes, is upregulated in microvessels in AD and plays a role in Aβ clearance (reviewed [47]). Whether endothelial activation predicts not only acute but also longer neurological complications (including dementia) in COVID-19 remains to be determined.

### Angiotensin-converting enzyme-2-mediated entry of SARS-CoV-2 into human cells and activation of the classical renin-angiotensin system

SARS-CoV-2 cell attachment and entry are initiated by binding of the virus to angiotensin-converting enzyme-2 (ACE-2) [120]. The expression of ACE-2 at the cell surface is therefore likely to be a critical determinant of viral tropism and pathobiology in COVID-19. ACE-2 is expressed in stem cell-derived neurons [121] and in neuronal and glial cells within the brain [33, 42], potentially enabling virus entry and spread through the cribriform plate by retrograde axonal transport along olfactory nerves [33], or from sensory fibres that pass from the lungs to the brain stem via the vagal nerve and nodose ganglion [4]. ACE-2 is also expressed in the temporal lobe and hippocampus—brain regions that are involved in cognition and memory and are affected in AD [122]. Neuronal uptake and spread within the brain were demonstrated in human ACE-2 transgenic mice infected with SARS-CoV-1 [123] and SARS-CoV-2 [124]. However, as noted above, ACE-2 is also highly expressed on endothelial cells and pericytes, and haematogenous spread followed by endothelial uptake or influx of infected peripheral immune cells are further possible routes of entry of virus into the brain.

**Fig. 2:**
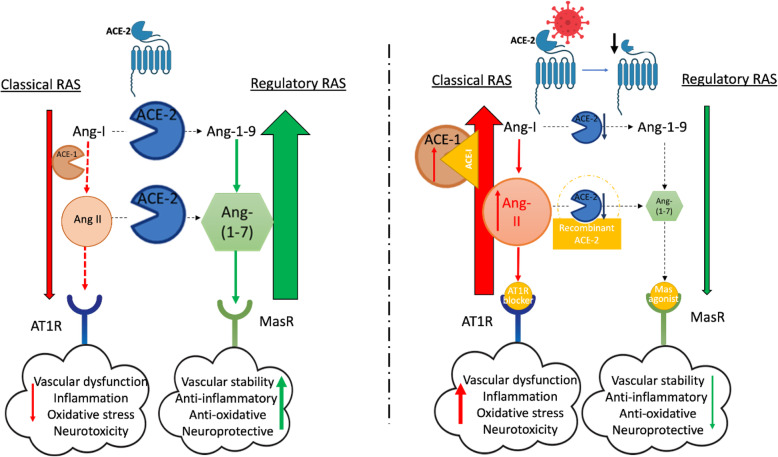
Altered balance between the classical and regulatory parts of the renin-angiotensin system (RAS) in COVID-19. **a** Ang-II is formed by the ACE-1-mediated cleavage of Ang-I. The binding of Ang-II to AT1R within the vasculature not only induces vasoconstriction but also affects vascular permeability and neurovascular coupling and promotes neuroinflammation and oxidative stress within the CNS. Under normal circumstances, these actions are counteracted by ACE-2 activity, which leads to the production of Ang-1-9 and Ang-(1-7) and the activation of MasR. **b** Internalisation or cleavage of membrane-bound ACE-2 following the binding and cell entry of SARS-CoV-2 virus leads to downregulation of the regulatory RAS and overaction of the classical RAS, driving vascular dysfunction, inflammation, oxidative stress and CNS injury in COVID-19

During SARS-Cov-1 infection, ACE-2 is cleaved from the cell surface [125] by ADAM-17 [126] during viral entry [127]. Although likely, it remains to be determined whether SARS-CoV-2 similarly results in loss of cell membrane-associated ACE-2. Ordinarily, ACE-2 is a key effector of the regulatory RAS that counters the actions of the classical RAS and reduces the risk of cardiovascular disease (reviewed [128, 129]), stroke [130–132], and dementia [133] (Fig. 2). ACE-2 is reduced in Alzheimer’s disease (AD) [42] and cognitive decline is pronounced in ACE-2 knock-out mice [134]. If SARS-CoV-2 entry leads to loss of ACE-2 (as in SARS), angiotensin II-mediated classical RAS activation would increase the risk of cerebrovascular and neurological disturbances in COVID-19 patients. This mechanism has also been proposed to explain other vascular and pulmonary manifestations of COVID-19 [135, 136]. Internalisation of ACE2, as a result of angiotensin II activation of angiotensin receptor type 1 (AT1R), may further exacerbate damage [137]. The available reservoir of ACE-2 may be an important determinant of clinical outcome in COVID-19. Studies in rodents indicate that ACE-2 expression declines with age and is lower in males [138–141]. In contrast, oestrogen upregulates ACE-2, which could help to protect pre-menopausal women against severe complications of COVID-19 [142, 143]. It may be relevant that most co-morbidities that increase the risk of complications in COVID-19, including hypertension, obesity, and diabetes, are associated overactivity of the classical RAS. Indeed ethnicity and genetic variations also influence baseline ACE-2 levels [144] and could provide a biological explanation as to why some ethnic groups are at higher risk of COVID-19 [145]. The hypothesis would explain why angiotensin receptor blockers (ARBs) and angiotensin-converting enzyme-1 inhibitors (ACE-Is), which downregulate the classical RAS by either blocking angiotensin II (Ang-II) signalling or Ang-II synthesis, respectively, and upregulate ACE-2, may reduce mortality in COVID-19 patients [146, 147].

An imbalance in RAS contributes to acute ARDS (reviewed [148]), which develops in a high proportion of the COVID-19 patients who develop viral pneumonia [149, 150]. ARDS patients show overactivity of the classical RAS [151] and reduction of ACE-2 [152, 153]. Increased Ang-II-mediated AT1R signalling, which drives inflammation [154], is likely to contribute to the inflammatory storm in severe COVID-19. Lung tissue damage in ARDS can be reduced by ARBs and ACEIs [155] and by administration of recombinant ACE-2 [152, 156]. ACE-2 catalyses the formation of Ang-(1-7), which binds to Mas receptor; both ACE-2 activation [157] and Ang-(1-7) activation of Mas receptor attenuate lung injury in ARDS [158]. Ang-(1-7) activates ERK1/2 signalling and modulates interleukin (IL)-10 expression, protecting against lung damage (reviewed [159]).

Recombinant soluble ACE-2 (rsACE-2) shows therapeutic promise in severe COVID-19 infection; administration was reported to reduce viral titre and serum Ang-II, elevate serum Ang-(1-7) levels and markedly reduce pro-inflammatory cytokines [160]. In addition to preventing viral binding, rsACE-2-mediated reduction in Ang-II is likely to prevent AT1R-mediated ADAM-17 cleavage of membrane-bound ACE-2 and restore balance in the RAS [161].

### Potential neurological consequences of increased classical renin-angiotensin system activation in COVID-19

The RAS is expressed and functions independently within the brain. Overactivation of the classical RAS, with elevated ACE-1 and Ang-II, has been demonstrated in post-mortem human brain tissue in AD [162–164]. Cerebroventricular infusion of Ang-II into adult Wistar rats promoted Aβ production and tau pathology [165, 166], and ARBs and ACEIs protect against cognitive decline and disease pathology in transgenic APP mouse models of AD (reviewed [167]). We previously reported that reduction of ACE-2 in brain tissue in AD correlated strongly with parenchymal Aβ and tau levels and with increased ACE-1 activity [42]. We and others have since shown that induction of ACE-2, or administration of Ang-(1-7) or peptide analogues, protects against Aβ-related cognitive decline in mice associated with reduced neuroinflammation and oxidative stress [133, 168, 169].

The RAS is a critical regulator of vascular function. Ang-II binds to AT1R on vascular smooth muscle cells [170, 171] to induce cerebral artery constriction [172], and on pericytes to cause constriction of microvessels [173, 174]. Ang-II also modulates BBB permeability: AT1R signalling induces leakiness in endothelial cell culture models of the BBB [175] and Ang-II infusion causes BBB leakiness in mice that can be reversed by adding a superoxide scavenger, indicating a role for oxidative stress [176]. Several mediators of BBB leakiness, including vascular endothelial growth factor and matrix metalloproteinase (MMP)-2 and MMP-9, are induced by Ang-II [177–179]. In mice, Ang-II was shown to impair neurovascular coupling (i.e. the blood flow response to increased neural activity) in the somatosensory cortex [180] and to interfere with cerebral autoregulation [181, 182]. The accumulation of ACE-1 in the extracellular matrix around cerebral arterioles (particularly in AD patients with cerebral amyloid angiopathy) suggests that locally produced (as well as circulatory) Ang-II participates in cerebrovascular dysfunction mediated by overactivation of the classical RAS [163].

Overactivation of the classical RAS may also reduce clearance of Aβ. Intra-mural peri-arterial drainage (IPAD) and para-vascular glymphatic channels have been implicated in the removal of Aβ from the brain [183–185]. The functioning of these drainage pathways depends on the polarised expression of aquaporin-4 in astrocytic endfeet [186], which is regulated by pericytes [187]. Focal absence of pericytes results in the redistribution of aquaporin-4 to the cell soma [51]. The RAS modulates aquaporin-4 expression in astrocytes [188], and Ang-II was shown to act via AT1R to reduce ACE-2 expression in astrocyte cultures [189]. These alterations in pericyte and astrocyte function are likely to impair the clearance of Aβ. It remains to be determined whether this occurs in COVID-19.

Neuroinflammation is strongly implicated in the development of AD. Genome-wide association studies have identified several inflammatory pathway genes as risk factors for AD [190, 191]. Complement [192] and inflammasome activation [193, 194] are likely to contribute to cerebrovascular dysfunction, neuronal toxicity, and the accumulation of Aβ and tau in AD. Ang-II activates the complement system [195, 196] and the NLRP3 inflammasome [197], and both complement [198] and inflammasome activation [11] have been proposed to contribute to neurological disease in COVID-19 patients. A recent in silico study implicated SARS-CoV-2 activation of Toll-like receptor 4 (TLR4) as a major contributor to the inflammatory response in COVID-19 [199]. Ang-II upregulates TLR4 [200], which is a critical determinant of Ang-II-mediated vascular remodelling [201]. Blocking of TLR4 signalling delayed the development of Ang-II-mediated hypertension in rats and was associated with a dramatic increase in ACE-2 [202]. Pericytes express high levels of TL4R, activated by free long-chain fatty-acids [203]. The spike protein of SARS-CoV-2 has been shown to bind to linoleic acid, affecting the conformation of the protein and possibly the binding of the virus to ACE-2 (pre-published study [204]). It may therefore be relevant that linoleic acid is reduced in both COVID-19 [205] and AD [206], potentially influencing the progression of both diseases. Ang-II also acts as a molecular switch regulating microglial phenotype—switching between an M1 (pro-inflammatory) and M2 (immunoregulatory) protective phagocytic phenotype [207] which is relevant to AD pathogenesis [208]. The role of microglia in neurological manifestations of COVID-19 has yet to be fully explored.

Ang-II-mediated endothelial activation promotes the binding and diapedesis of leukocytes across the BBB; these effects are mitigated by Ang-1-7 [209]. Pericytes too have immune-regulating properties (reviewed in [210]) and their localisation within the cerebral vasculature suggests that they may serve a ‘gate-keeper’ role in regulating immune cell infiltration. Although pericytes express ACE-2, it remains to be established whether they are targeted by SARS-CoV-2. Because of the pivotal role of pericytes in regulation of cerebrovascular function (and perhaps immune cell infiltration), it is likely that virus-induced pericyte damage would compromise cerebral perfusion, BBB integrity, and immune regulation. In addition to vascular effects, angiotensin peptides derived from Ang-II, including Ang-IV and Ang-(1-7), have neuromodulatory [211] (reviewed [212]) and neuroprotective properties (reviewed [213]). Ang-(1-7) activation of Mas (regulatory RAS) receptor and Ang-IV activation of the c-Met and insulin-regulated aminopeptidase receptors (reviewed [214]) limit tissue damage in models of stroke (reviewed [215, 216]). Similarly, ACE-2 activation and/or Ang-(1-7) infusion prevents cognitive decline and disease pathology in animal models of Aβ accumulation [133, 169, 217, 218], independent of changes in blood pressure. There is therefore a wide range of mechanisms through which reduced regulatory RAS signalling may exacerbate brain damage in COVID-19.

### Is APOE ε4, an established risk factor for AD and vascular dysfunction, also a risk factor for COVID-19?

*APOE* polymorphism greatly influences the risk of developing AD: the risk is increased with *APOE* ε4 and decreased with *APOE* ε2 [219, 220]. The physiological roles of the encoded apolipoprotein (apolipoprotein E, ApoE) have still not been fully defined. Recent studies indicate that possession of *APOE* ε4 is associated with cerebrovascular dysfunction, including BBB leakiness and pericyte degeneration [221] and cerebral amyloid angiopathy with capillary involvement [222]. A recent UK study reported that there was a higher prevalence of COVID-19 in people who were carriers of *APOE* ε4 [223]. We previously showed that *APOE* ε4 individuals also have lowest ACE-2 activity [42]. Pericyte expression of *APOE* ε4 was reported to promote BBB leakiness because of deficient basement membrane formation [224]. Moreover, possession of *APOE* ε4 is associated with reduced cerebral blood flow and increased subcortical ischaemic white matter damage [225, 226], as well as neuroinflammation in AD (reviewed [227]). Future studies should aim to clarify the relationships between *APOE* ε4, COVID-19, and cerebral vascular dysfunction and AD.

### Potential upregulation of ADAM-17 in COVID-19

ACE-2 is cleaved by ADAM-17 upon SARS-Cov-1 entry into cells [125–127]. It seems likely that this also occurs upon SARS-Cov-2 cell entry, although the data are not yet available. Ang-II-mediated activation of ADAM-17 and shedding of ACE-2 points to a positive feedback loop in which increased Ang-II level is associated with loss of ACE-2 [228]. Yet, it is worthy of note that ADAM-17 cleaves many cell-associated proteins that are required for proper function of the vasculature including the ApoE receptor, low-density lipoprotein receptor-related protein 1 (LRP-1), involved in transendothelial clearance of Aβ (facilitated by ApoE), and PDGFRβ, needed for maintenance of pericytes. Upregulation of ADAM-17 may therefore potentially exacerbate vascular dysfunction in COVID-19. ADAM-17 also acts as one of the α-secretases and cleaves APP, preventing the generation of Aβ (reviewed [229]). The complex divergent roles of ADAM-17 in AD and potentially COVID-19 require further investigation.

### Clinical management, clinical trials and possible future targets for therapeutic intervention in COVID-19 patients

In severe COVID-19, patients present with pneumonia and those most severely affected develop ARDS with features of septic shock and multiple organ failure, requiring oxygen treatment and/or mechanical ventilation. Infection-induced inflammatory and vascular changes associated with coagulopathy and thrombosis, including venous thromboembolism (VTE), DIC and thrombotic microangiopathy with thromboembolic microvascular complications, are common complications of severe COVID-19, as illustrated by reports of VTE in 25–27% hospitalised patients [230, 231]. The International Society on Thrombosis and Hemostasis (ISTH) recommends measuring levels of d-dimer, prothrombin time, partial thromboplastin time and platelet count, in hospitalised COVID-19 patients [232]. A retrospective study in Wuhan, China, at the beginning of the pandemic, found that mortality rates were lower in patients given low-molecular weight heparin [233]. Clinical management of severe COVID-19 patients now routinely includes low-dose sub-cutaneous heparin [233] and/or thrombotic prophylaxis [234], unless patients are at increased risk of bleeding.

Lymphopenia with marked loss of regulatory T and B cells and natural killer cells, reduction in monocytes, eosinophils and basophils, and an increase in neutrophils are typical in severe COVID-19 (reviewed [235]). There are also elevated levels of pro-inflammatory cytokines, sometimes marked (a so-called cytokine storm). Convalescent plasma [236] and plasma exchange [237, 238] improve survival rates in severe disease, and immunomodulatory therapies such as tocilizumab, a monoclonal antibody against the IL-6 receptor [239], and sarilumab, an IL-6 receptor antagonist [240], may offer protection and are currently undergoing clinical trials. Neutralising antibodies targeting other pro-inflammatory cytokines (IL-1, IL-17) may also offer protection, as too may potential inhibitors of complement system activation. Intravenous transplantation of mesenchymal stems cells was shown to improve the outcome of COV-19 patients with pneumonia in 7 COVID-19 patients in Beijing, China [241]. It seems possible that mesenchymal stem cells might also ameliorate brain injury in severe COVID-19, given their immunomodulatory and anti-inflammatory properties, and ability to attenuate BBB damage and neuroinflammation after cerebral ischaemia [242–244].

There is continued debate on the role of systemic or inhaled corticosteroids in COVID-19 patients. Although earlier studies indicated a lack of benefit from corticosteroids [245], a randomised clinical trial reported by the RECOVERY collaborative group, Oxford, UK, found that systemic dexamethasone reduced mortality in severely affected COVID-19 patients [246]. Inhaled steroids had previously been shown to reduce inflammation and tissue injury in ARDS [247] (reviewed [248]). In addition to their inherent anti-inflammatory properties, steroids may have anti-viral properties [249]. Ciclesonide, an inhaled corticosteroid, was shown to suppress replication of MERS-CoV, SARS-CoV and SARS-CoV-2 in vitro [250].

The expression and activity of interferon-β (IFN-β), an endogenous protein with anti-viral and anti-inflammatory properties, is impaired in COVID-19 [251]. Interferon inhibits SARS-CoV-2 replication in vitro [252]. In a Phase II clinical trial, IFN-β in combination with anti-viral drugs shortened the duration of viral shedding and length of hospital admission [253]. A pharmaceutical company based in the UK, Synairgen, reported a lower risk of requiring ventilation, and reduction in mortality by about 79%, in a Phase II clinical trial of SNG001, an inhaled form of IFN-β (these data are currently unpublished).

Since ACE-2 is a receptor for SARS-CoV-2, and ACEIs and ARBs are predicted to increase ACE-2 expression, it was initially feared that the use of these drugs might exacerbate COVID-19 [254]. Recent meta-analyses have instead suggested that RAS-targeting medications are protective in COVID-19 [146, 147, 255]. This is likely to be a due to the protective role of ACE-2 in lowering or preventing overactivation of the classical RAS and minimising consequent Ang-II-mediated ischaemic and inflammatory damage, as outlined above. Several clinical trials have been registered with the National Institutes of Health (NIH) to test ARBs such as losartan in COVID-19 patients: NCT04335123, NCT04312009 and NCT04311177. Two studies are also investigating the impact of discontinuation of ACE-Is on COVID-19 (EudraCT numbers 2020-001544-26 and 2020-001206-35). Boosting the regulatory arm of the RAS may ameliorate COVID-19 because of the protective effects of ACE-2 and Ang-(1-7); interventional trials with recombinant human ACE2 (rhACE2) and Ang-(1–7) have also been registered (NCT04287686 and NCT04332666, respectively), although the rhACE2 study seeking to recruit people between the ages 18–80 years in China has since been withdrawn. A further rhACE2 study (2020-001172-15) is registered on the EU Clinical trials register. Other strategies being explored include several studies seeking to inhibit TMPRSS2. Studies that could include TL4R blockers and ADAM-17 inhibitors might also be worthy of future study. For a comprehensive review of the pharmacological targets that are currently being investigated as potential interventions and treatments in COVID-19, the reader should refer to recent reviews [256–258].

## Conclusion

Cerebral vascular disease is emerging as a major complication of severe COVID-19. This is likely to cause lasting brain damage and to increase the risk of stroke and vascular cognitive impairment. Several of the metabolic abnormalities that affect COVID-19 patients may also increase the risk of developing AD. Dementia and COVID-19 share many co-morbidities and risk factors, including age, gender, hypertension, diabetes, obesity and possession of *APOE* ε4—most of which are associated with an overactive RAS, cerebrovascular dysfunction and neuroinflammation. These shared co-morbidities and similar mechanisms may also explain the high incidence and increased rates of mortality amongst people with dementia [59, 259, 260]. There is urgent need for research to better understand the pathogenesis of neurological disturbances in COVID-19, some of which have probably been covert and the prevalence of which may be considerably underestimated. This understanding is essential to establish the long-term consequences from the disease (including the potential for increased risk of dementia in some cases) and to identify means of preventing or ameliorating the brain damage.

## Data Availability

Data sharing is not applicable to this article as no datasets were generated or analysed during the current study.

## Abbreviations

Aβ: Amyloid-β
ACE: Angiotensin-converting enzyme
ACE-I: Angiotensin-converting enzyme-1 inhibitor
AD: Alzheimer’s disease
ApoE: Apolipoprotein E
APP: Amyloid-β precursor protein
Ang-II: Angiotensin II
ARB: Angiotensin receptor blocker
ARDS: Acute respiratory distress syndrome
AT1R: Angiotensin type 1 receptor
BBB: Blood-brain barrier
BSG: Basigin
CNS: Central nervous system
CVD: Cerebrovascular disease
DIC: Disseminated intravascular coagulation
IFN-β: Interferon-β
ICH: Intracerebral haemorrhage
IL: Interleukin
LRP-1: Low-density lipoprotein receptor-related protein 1
MMP: Matrix metalloproteinase
MER: Middle East respiratory syndrome
NRP1: Neuropilin
PDGFRβ: Platelet-derived growth factor-β
PET: Positron emission tomography
RAS: Renin-angiotensin system
SARS: Severe acute respiratory syndrome
SARS-CoV-2: Severe acute respiratory syndrome coronavirus-2
SVD: Small vessel disease
TLR4: Toll-like receptor 4

## References

[1] Desforges M, Le Coupanec A, Dubeau P, Bourgouin A, Lajoie L, Dubé M, Talbot PJ. Human coronaviruses and other respiratory viruses: underestimated opportunistic pathogens of the central nervous system? Viruses. 2019;12(1):14. 10.3390/v12010014. 10.3390/v12010014 PMC7020001 31861926

[2] Glass WG (2004). Mechanisms of host defense following severe acute respiratory syndrome-coronavirus (SARS-CoV) pulmonary infection of mice. J Immunol.

[3] Gu J (2005). Multiple organ infection and the pathogenesis of SARS. J Exp Med.

[4] Li YC, Bai WZ, Hashikawa T (2020). The neuroinvasive potential of SARS-CoV2 may play a role in the respiratory failure of COVID-19 patients. J Med Virol.

[5] Ellul MA, Benjamin L, Singh B, Lant S, Michael BD, Easton A, Kneen R, Defres S, Sejvar J, Solomon T. Neurological associations of COVID-19. Lancet Neurol. 2020;19(9):767–83. 10.1016/S1474-4422(20)30221-0. 10.1016/S1474-4422(20)30221-0 PMC7332267 32622375

[6] Mao L, Jin H, Wang M, Hu Y, Chen S, He Q, Chang J, Hong C, Zhou Y, Wang D, Miao X, Li Y, Hu B. Neurologic Manifestations of Hospitalized Patients With Coronavirus Disease 2019 in Wuhan, China. JAMA Neurol. 2020;77(6):683–90. 10.1001/jamaneurol.2020.1127. 10.1001/jamaneurol.2020.1127 PMC7149362 32275288

[7] Helms J (2020). Neurologic features in severe SARS-CoV-2 infection. N Engl J Med.

[8] Varatharaj A, Thomas N, Ellul MA, Davies NWS, Pollak TA, Tenorio EL, Sultan M, Easton A, Breen G, Zandi M, Coles JP, Manji H, Al-Shahi Salman R, Menon DK, Nicholson TR, Benjamin LA, Carson A, Smith C, Turner MR, Solomon T, Kneen R, Pett SL, Galea I, Thomas RH, Michael BD. CoroNerve Study Group. Neurological and neuropsychiatric complications of COVID-19 in 153 patients: a UK-wide surveillance study. Lancet Psychiatry. 2020;7(10):875–82. 10.1016/S2215-0366(20)30287-X. 10.1016/S2215-0366(20)30287-X PMC7316461 32593341

[9] Lu Y, Li X, Geng D, Mei N, Wu PY, Huang CC, Jia T, Zhao Y, Wang D, Xiao A, Yin B. Cerebral micro-structural changes in COVID-19 patients - an MRI-based 3-month follow-up study. EClinicalMed, 2020;25:100484. 10.1016/j.eclinm.2020.100484. 10.1016/j.eclinm.2020.100484 PMC7396952 32838240

[10] Pereira A (2020). Long-term neurological threats of COVID-19: a call to update the thinking about the outcomes of the coronavirus pandemic. Front Neurol.

[11] Heneka MT (2020). Immediate and long-term consequences of COVID-19 infections for the development of neurological disease. Alzheimers Res Ther.

[12] Lindlau A (2015). Predictors of hippocampal atrophy in critically ill patients. Eur J Neurol.

[13] Iwashyna TJ (2010). Long-term cognitive impairment and functional disability among survivors of severe sepsis. JAMA.

[14] Widmann CN, Heneka MT (2014). Long-term cerebral consequences of sepsis. Lancet Neurol.

[15] Girard TD (2018). Clinical phenotypes of delirium during critical illness and severity of subsequent long-term cognitive impairment: a prospective cohort study. Lancet Respir Med.

[16] Sasannejad C, Ely EW, Lahiri S (2019). Long-term cognitive impairment after acute respiratory distress syndrome: a review of clinical impact and pathophysiological mechanisms. Crit Care.

[17] Paterson RW, Brown RL, Benjamin L, Nortley R, Wiethoff S, Bharucha T, Jayaseelan DL, Kumar G, Raftopoulos RE, Zambreanu L, Vivekanandam V, Khoo A, Geraldes R, Chinthapalli K, Boyd E, Tuzlali H, Price G, Christofi G, Morrow J, McNamara P, McLoughlin B, Lim ST, Mehta PR, Levee V, Keddie S, Yong W, Trip SA, Foulkes AJM, Hotton G, Miller TD, Everitt AD, Carswell C, Davies NWS, Yoong M, Attwell D, Sreedharan J, Silber E, Schott JM, Chandratheva A, Perry RJ, Simister R, Checkley A, Longley N, Farmer SF, Carletti F, Houlihan C, Thom M, Lunn MP, Spillane J, Howard R, Vincent A, Werring DJ, Hoskote C, Jäger HR, Manji H, Zandi MS. The emerging spectrum of COVID-19 neurology: clinical, radiological and laboratory findings. Brain. 2020;143(10):3104–20. 10.1093/brain/awaa240. 10.1093/brain/awaa240 PMC7454352 32637987

[18] Hernández-Fernández F, Sandoval Valencia H, Barbella-Aponte RA, Collado-Jiménez R, Ayo-Martín Ó, Barrena C, Molina-Nuevo JD, García-García J, Lozano-Setién E, Alcahut-Rodriguez C, Martínez-Martín Á, Sánchez-López A, Segura T. Cerebrovascular disease in patients with COVID-19: neuroimaging, histological and clinical description. Brain. 2020;143(10):3089–103. 10.1093/brain/awaa239. 10.1093/brain/awaa239 PMC7454411 32645151

[19] Benussi A, Pilotto A, Premi E, Libri I, Giunta M, Agosti C, Alberici A, Baldelli E, Benini M, Bonacina S, Brambilla L, Caratozzolo S, Cortinovis M, Costa A, Cotti Piccinelli S, Cottini E, Cristillo V, Delrio I, Filosto M, Gamba M, Gazzina S, Gilberti N, Gipponi S, Imarisio A, Invernizzi P, Leggio U, Leonardi M, Liberini P, Locatelli M, Masciocchi S, Poli L, Rao R, Risi B, Rozzini L, Scalvini A, Schiano di Cola F, Spezi R, Vergani V, Volonghi I, Zoppi N, Borroni B, Magoni M, Pezzini A, Padovani A. Clinical characteristics and outcomes of inpatients with neurologic disease and COVID-19 in Brescia, Lombardy, Italy. Neurology. 2020;95(7):e910–20. 10.1212/WNL.0000000000009848. 10.1212/WNL.0000000000009848 32444493

[20] Aggarwal G, Lippi G, Michael Henry B (2020). Cerebrovascular disease is associated with an increased disease severity in patients with Coronavirus Disease 2019 (COVID-19): a pooled analysis of published literature. Int J Stroke.

[21] Oxley TJ (2020). Large-vessel stroke as a presenting feature of Covid-19 in the young. N Engl J Med.

[22] Reddy ST (2020). Cerebrovascular disease in patients with COVID-19: a review of the literature and case series. Case Rep Neurol.

[23] Teuwen LA (2020). COVID-19: the vasculature unleashed. Nat Rev Immunol.

[24] Varga Z (2020). Endothelial cell infection and endotheliitis in COVID-19. Lancet.

[25] Solomon IH, Normandin E, Bhattacharyya S, Mukerji SS, Keller K, Ali AS, Adams G, Hornick JL, Padera RF Jr, Sabeti P. Neuropathological Features of Covid-19. N Engl J Med. 2020;383(10):98–92. 10.1056/NEJMc2019373. 10.1056/NEJMc2019373 PMC7304421 32530583

[26] Coolen T, Lolli V, Sadeghi N, Rovai A, Trotta N, Taccone FS, Creteur J, Henrard S, Goffard JC, Dewitte O, Naeije G, Goldman S, De Tiège X. Early postmortem brain MRI findings in COVID-19 non-survivors. Neurology. 2020;95(14):e2016–27. 10.1212/WNL.0000000000010116. 10.1212/WNL.0000000000010116 32546654

[27] Kanberg N, Ashton NJ, Andersson LM, Yilmaz A, Lindh M, Nilsson S, Price RW, Blennow K, Zetterberg H, Gisslén M. Neurochemical evidence of astrocytic and neuronal injury commonly found in COVID-19. Neurology. 2020;95(12):e1754–9. 10.1212/WNL.0000000000010111. 10.1212/WNL.0000000000010111 32546655

[28] Matschke J (2020). Neuropathology of patients with COVID-19 in Germany: a post-mortem case series. Lancet Neurol.

[29] Moriguchi T (2020). A first case of meningitis/encephalitis associated with SARS-Coronavirus-2. Int J Infect Dis.

[30] Paniz-Mondolfi A (2020). Central nervous system involvement by severe acute respiratory syndrome coronavirus-2 (SARS-CoV-2). J Med Virol.

[31] Hamming I (2004). Tissue distribution of ACE2 protein, the functional receptor for SARS coronavirus. A first step in understanding SARS pathogenesis. J Pathol.

[32] Chen L (2020). The ACE2 expression in human heart indicates new potential mechanism of heart injury among patients infected with SARS-CoV-2. Cardiovasc Res.

[33] Baig AM (2020). Evidence of the COVID-19 virus targeting the CNS: tissue distribution, host-virus interaction, and proposed neurotropic mechanisms. ACS Chem Neurosci.

[34] Li MY (2020). Expression of the SARS-CoV-2 cell receptor gene ACE2 in a wide variety of human tissues. Infect Dis Poverty.

[35] Qi F (2020). Single cell RNA sequencing of 13 human tissues identify cell types and receptors of human coronaviruses. Biochem Biophys Res Commun.

[36] Sungnak W (2020). SARS-CoV-2 entry factors are highly expressed in nasal epithelial cells together with innate immune genes. Nat Med.

[37] Chen R, et al. The spatial and cell-type distribution of SARS-CoV-2 receptor ACE2 in human and mouse brain. BioRxiv. 2020; 10.1101/2020.04.07.030650. 10.3389/fneur.2020.573095 PMC7855591 33551947

[38] Song E, et al. Neuroinvasion of SARS-CoV-2 in human and mouse brain. BioRxiv. 2020; 10.1101/2020.06.25.169946. 10.1084/jem.20202135 PMC7808299 33433624

[39] Zhang BZ, Chu H, Han S, Shuai H, Deng J, Hu YF, Gong HR, Lee AC, Zou Z, Yau T, Wu W, Hung IF, Chan JF, Yuen KY, Huang JD. SARS-CoV-2 infects human neural progenitor cells and brain organoids. Cell Res. 2020;30(10):928–31. 10.1038/s41422-020-0390-x. 10.1038/s41422-020-0390-x PMC7399356 32753756

[40] Kase Y, Okano H. Expression of ACE2 and a viral virulence-regulating factor CCN family member 1 in human iPSC-derived neural cells: implications for COVID-19-related CNS disorders. Inflamm Regen. 2020;40:32. 10.1186/s41232-020-00143-6. 10.1186/s41232-020-00143-6 PMC7485212 32934757

[41] Ramani, A., et al., SARS-CoV-2 targets cortical neurons of 3D human brain organoids and shows neurodegeneration-like effects BioRxiv, 2020. doi: 10.1101/2020.05.20.106575doi.

[42] Kehoe PG (2016). Angiotensin-converting enzyme 2 is reduced in Alzheimer’s disease in association with increasing amyloid-beta and tau pathology. Alzheimers Res Ther.

[43] He, L., et al., Pericyte-specific vascular expression of SARS-CoV-2 receptor ACE2 – implications for microvascular inflammation and hypercoagulopathy in COVID-19 patients. prepublished bioRxiv doi: 10.1101/2020.05.11.0885002020.

[44] Wang, K., et al., SARS-CoV-2 invades host cells via a novel route: CD147-spike protein. doi: 10.1101/2020.03.14.9883452020. 10.1038/s41392-020-00426-x PMC7714896 33277466

[45] Cantuti-Castelvetri L, et al. Neuropilin-1 facilitates SARS-CoV-2 cell entry and provides a possible pathway into the central nervous system. BioRxix. 2020; 10.1101/2020.06.07.137802.

[46] Iadecola C, Anrather J, Kamel H. Effects of COVID-19 on the Nervous System. Cell. 2020;183(1):16-27.e1. 10.1016/j.cell.2020.08.028. 10.1016/j.cell.2020.08.028 PMC7437501 32882182

[47] Zenaro E, Piacentino G, Constantin G (2017). The blood-brain barrier in Alzheimer’s disease. Neurobiol Dis.

[48] Attwell D (2016). What is a pericyte?. J Cereb Blood Flow Metab.

[49] Sweeney MD, Ayyadurai S, Zlokovic BV (2016). Pericytes of the neurovascular unit: key functions and signaling pathways. Nat Neurosci.

[50] Hall CN (2014). Capillary pericytes regulate cerebral blood flow in health and disease. Nature.

[51] Armulik A (2010). Pericytes regulate the blood-brain barrier. Nature.

[52] Ribatti D, Nico B, Crivellato E (2011). The role of pericytes in angiogenesis. Int J Dev Biol.

[53] Ziegler CGK (2020). SARS-CoV-2 receptor ACE2 is an interferon-stimulated gene in human airway epithelial cells and is detected in specific cell subsets across tissues. Cell.

[54] He L (2018). Single-cell RNA sequencing of mouse brain and lung vascular and vessel-associated cell types. Sci Data.

[55] He L (2016). Analysis of the brain mural cell transcriptome. Sci Rep.

[56] Vanlandewijck M (2018). A molecular atlas of cell types and zonation in the brain vasculature. Nature.

[57] Cardot-Leccia N, Hubiche T, Dellamonica J, Burel-Vandenbos F, Passeron T. Pericyte alteration sheds light on micro-vasculopathy in COVID-19 infection. Intensive Care Med. 2020;46(9):1777–8. 10.1007/s00134-020-06147-7. 10.1007/s00134-020-06147-7 PMC7291173 32533198

[58] Williamson EJ (2020). Factors associated with COVID-19-related death using OpenSAFELY. Nature.

[59] Atkins JL, Masoli JAH, Delgado J, Pilling LC, Kuo CL, Kuchel GA, Melzer D. Preexisting Comorbidities Predicting COVID-19 and Mortality in the UK Biobank Community Cohort. J Gerontol A Biol Sci Med Sci. 2020;75(11):2224–30. 10.1093/gerona/glaa183. 10.1093/gerona/glaa183 PMC7454409 32687551

[60] Mok VCT, Pendlebury S, Wong A, Alladi S, Au L, Bath PM, Biessels GJ, Chen C, Cordonnier C, Dichgans M, Dominguez J, Gorelick PB, Kim S, Kwok T, Greenberg SM, Jia J, Kalaria R, Kivipelto M, Naegandran K, Lam LCW, Lam BYK, Lee ATC, Markus HS, O'Brien J, Pai MC, Pantoni L, Sachdev P, Skoog I, Smith EE, Srikanth V, Suh GH, Wardlaw J, Ko H, Black SE, Scheltens P. Tackling challenges in care of Alzheimer's disease and other dementias amid the COVID-19 pandemic, now and in the future. Alzheimers Dement. 2020;16(11):1571–81. 10.1002/alz.12143. 10.1002/alz.12143 PMC7436526 32789951

[61] Pendlebury ST, Rothwell PM, Oxford Vascular S (2019). Incidence and prevalence of dementia associated with transient ischaemic attack and stroke: analysis of the population-based Oxford Vascular study. Lancet Neurol.

[62] Mijajlovic MD (2017). Post-stroke dementia - a comprehensive review. BMC Med.

[63] Staekenborg SS (2008). Small vessel versus large vessel vascular dementia: risk factors and MRI findings. J Neurol.

[64] Sudlow CL, Warlow CP (1997). Comparable studies of the incidence of stroke and its pathological types: results from an international collaboration. International Stroke Incidence Collaboration. Stroke.

[65] Gorelick PB (2011). Vascular contributions to cognitive impairment and dementia: a statement for healthcare professionals from the American Heart Association/American Stroke Association. Stroke.

[66] Wardlaw JM, Smith C, Dichgans M (2019). Small vessel disease: mechanisms and clinical implications. Lancet Neurol.

[67] Ostergaard L (2016). Cerebral small vessel disease: capillary pathways to stroke and cognitive decline. J Cereb Blood Flow Metab.

[68] Brun G, Hak JF, Coze S, Kaphan E, Carvelli J, Girard N, Stellmann JP. COVID-19-White matter and globus pallidum lesions: demyelination or small-vessel vasculitis? Neurol Neuroimmunol Neuroinflamm. 2020;7(4):e777. 10.1212/NXI.0000000000000777. 10.1212/NXI.0000000000000777 PMC7286652 32444427

[69] Reichard RR (2020). Neuropathology of COVID-19: a spectrum of vascular and acute disseminated encephalomyelitis (ADEM)-like pathology. Acta Neuropathol.

[70] Lang M, Buch K, Li MD, Mehan WA Jr, Lang AL, Leslie-Mazwi TM, Rincon SP. Leukoencephalopathy Associated with Severe COVID-19 Infection: Sequela of Hypoxemia? AJNR Am J Neuroradiol. 2020;41(9):1641–5. 10.3174/ajnr.A6671. 10.3174/ajnr.A6671 PMC7583106 32586959

[71] Bennett IJ, Madden DJ (2014). Disconnected aging: cerebral white matter integrity and age-related differences in cognition. Neuroscience.

[72] Ystad M (2011). Cortico-striatal connectivity and cognition in normal aging: a combined DTI and resting state fMRI study. Neuroimage.

[73] Wardlaw JM, Smith C, Dichgans M (2013). Mechanisms of sporadic cerebral small vessel disease: insights from neuroimaging. Lancet Neurol.

[74] Montagne A (2018). Pericyte degeneration causes white matter dysfunction in the mouse central nervous system. Nat Med.

[75] Petersen MA (2017). Fibrinogen activates BMP signaling in oligodendrocyte progenitor cells and inhibits remyelination after vascular damage. Neuron.

[76] Keable A (2016). Deposition of amyloid β in the walls of human leptomeningeal arteries in relation to perivascular drainage pathways in cerebral amyloid angiopathy. Biochim Biophys Acta.

[77] Weller RO (2008). Perivascular drainage of amyloid-β peptides from the brain and its failure in cerebral amyloid angiopathy and Alzheimer’s disease. Brain Pathol.

[78] Brown R (2018). Understanding the role of the perivascular space in cerebral small vessel disease. Cardiovasc Res.

[79] Mestre H (2017). Perivascular spaces, glymphatic dysfunction, and small vessel disease. Clin Sci (Lond).

[80] Charidimou A (2017). MRI-visible perivascular spaces in cerebral amyloid angiopathy and hypertensive arteriopathy. Neurology.

[81] Charidimou A (2014). White matter perivascular spaces are related to cortical superficial siderosis in cerebral amyloid angiopathy. Stroke.

[82] Charidimou A (2014). White matter perivascular spaces: an MRI marker in pathology-proven cerebral amyloid angiopathy?. Neurology.

[83] Czarnowska E, Karwatowska-Prokopczuk E (1995). Ultrastructural demonstration of endothelial glycocalyx disruption in the reperfused rat heart. Involvement of oxygen free radicals. Basic Res Cardiol.

[84] Ishiharajima S (1986). Early membrane damage during ischemia in rat heart. Exp Mol Pathol.

[85] Attems J, Jellinger KA (2014). The overlap between vascular disease and Alzheimer's disease--lessons from pathology. BMC Med.

[86] Brun A, Englund E (1986). A white matter disorder in dementia of the Alzheimer type: a pathoanatomical study. Ann Neurol.

[87] Love S, Miners JS (2016). Cerebrovascular disease in ageing and Alzheimer’s disease. Acta Neuropathol.

[88] Love S, Miners JS (2017). Small vessel disease, neurovascular regulation and cognitive impairment: post-mortem studies reveal a complex relationship, still poorly understood. Clin Sci (Lond).

[89] Montagne A (2015). Blood-brain barrier breakdown in the aging human hippocampus. Neuron.

[90] Nation DA (2019). Blood-brain barrier breakdown is an early biomarker of human cognitive dysfunction. Nat Med.

[91] Lee S (2016). White matter hyperintensities are a core feature of Alzheimer’s disease: evidence from the dominantly inherited Alzheimer network. Ann Neurol.

[92] Lee S (2018). White matter hyperintensities and the mediating role of cerebral amyloid angiopathy in dominantly-inherited Alzheimer’s disease. Plos One.

[93] Benzinger TL (2013). Regional variability of imaging biomarkers in autosomal dominant Alzheimer’s disease. Proc Natl Acad Sci U S A.

[94] Nortley R, Korte N, Izquierdo P, Hirunpattarasilp C, Mishra A, Jaunmuktane Z, Kyrargyri V, Pfeiffer T, Khennouf L, Madry C, Gong H, Richard-Loendt A, Huang W, Saito T, Saido TC, Brandner S, Sethi H, Attwell D. Amyloid β oligomers constrict human capillaries in Alzheimer's disease via signaling to pericytes. Science. 2019;365(6450):eaav9518. 10.1126/science.aav9518. 10.1126/science.aav9518. PMC6658218 31221773

[95] Hald ES, Timm CD, Alford PW. Amyloid Beta Influences Vascular Smooth Muscle Contractility and Mechanoadaptation. J Biomech Eng. 2016;138(11). 10.1115/1.4034560. 10.1115/1.4034560 27590124

[96] Marco S, Skaper SD (2006). Amyloid β-peptide1-42 alters tight junction protein distribution and expression in brain microvessel endothelial cells. Neurosci Lett.

[97] Tai LM (2010). Amyloid-β-induced occludin down-regulation and increased permeability in human brain endothelial cells is mediated by MAPK activation. J Cell Mol Med.

[98] Biron KE (2011). Amyloid triggers extensive cerebral angiogenesis causing blood brain barrier permeability and hypervascularity in Alzheimer’s disease. Plos One.

[99] Hartz AM (2012). Amyloid-β contributes to blood-brain barrier leakage in transgenic human amyloid precursor protein mice and in humans with cerebral amyloid angiopathy. Stroke.

[100] Halliday MR (2016). Accelerated pericyte degeneration and blood-brain barrier breakdown in apolipoprotein E4 carriers with Alzheimer’s disease. J Cereb Blood Flow Metab.

[101] Sengillo JD (2013). Deficiency in mural vascular cells coincides with blood-brain barrier disruption in Alzheimer’s disease. Brain Pathol.

[102] Ding R, Hase Y, Ameen-Ali KE, Ndung'u M, Stevenson W, Barsby J, Gourlay R, Akinyemi T, Akinyemi R, Uemura MT, Polvikoski T, Mukaetova-Ladinska E, Ihara M, Kalaria RN. Loss of capillary pericytes and the blood-brain barrier in white matter in poststroke and vascular dementias and Alzheimer's disease. Brain Pathol. 2020;30(6):1087–101. 10.1111/bpa.12888. 10.1111/bpa.12888 PMC8018063 32705757

[103] Sagare AP (2013). Pericyte loss influences Alzheimer-like neurodegeneration in mice. Nat Commun.

[104] Miners JS, Schulz I, Love S (2018). Differing associations between Aβ accumulation, hypoperfusion, blood-brain barrier dysfunction and loss of PDGFRB pericyte marker in the precuneus and parietal white matter in Alzheimer’s disease. J Cereb Blood Flow Metab.

[105] Verbeek MM (1997). Rapid degeneration of cultured human brain pericytes by amyloid β protein. J Neurochem.

[106] Rabin JS (2019). Vascular risk and β-amyloid are synergistically associated with cortical tau. Ann Neurol.

[107] Kim HJ (2018). Assessment of extent and role of tau in subcortical vascular cognitive impairment using 18F-AV1451 positron emission tomography imaging. JAMA Neurol.

[108] Wen Y (2004). Transient cerebral ischemia induces site-specific hyperphosphorylation of tau protein. Brain Res.

[109] Qiu L (2016). Chronic cerebral hypoperfusion enhances tau hyperphosphorylation and reduces autophagy in Alzheimer's disease mice. Sci Rep.

[110] Burkhart KK (1998). Alterations in tau phosphorylation in rat and human neocortical brain slices following hypoxia and glucose deprivation. Exp Neurol.

[111] Liu CC, Yamazaki Y, Heckman MG, Martens YA, Jia L, Yamazaki A, Diehl NN, Zhao J, Zhao N, DeTure M, Davis MD, Felton LM, Qiao W, Li Y, Li H, Fu Y, Wang N, Wren M, Aikawa T, Holm ML, Oue H, Linares C, Allen M, Carrasquillo MM, Murray ME, Petersen RC, Ertekin-Taner N, Dickson DW, Kanekiyo T, Bu G. Tau and apolipoprotein E modulate cerebrovascular tight junction integrity independent of cerebral amyloid angiopathy in Alzheimer's disease. Alzheimers Dement. 2020;16(10):1372–83. 10.1002/alz.12104. 10.1002/alz.12104 PMC8103951 32827351

[112] Bennett RE (2018). Tau induces blood vessel abnormalities and angiogenesis-related gene expression in P301L transgenic mice and human Alzheimer’s disease. Proc Natl Acad Sci U S A.

[113] Park L (2020). Tau induces PSD95-neuronal NOS uncoupling and neurovascular dysfunction independent of neurodegeneration. Nat Neurosci.

[114] Bourassa P (2020). Brain mural cell loss in the parietal cortex in Alzheimer’s disease correlates with cognitive decline and TDP-43 pathology. Neuropathol Appl Neurobiol.

[115] Thammisetty SS (2018). Age-related deregulation of TDP-43 after stroke enhances NF-kappaB-mediated inflammation and neuronal damage. J Neuroinflammation.

[116] Swirski M (2014). Evaluating the relationship between amyloid-β and α-synuclein phosphorylated at Ser129 in dementia with Lewy bodies and Parkinson’s disease. Alzheimers Res Ther.

[117] Pietronigro E, Zenaro E, Constantin G (2016). Imaging of leukocyte trafficking in Alzheimer’s disease. Front Immunol.

[118] Zenaro E (2015). Neutrophils promote Alzheimer’s disease-like pathology and cognitive decline via LFA-1 integrin. Nat Med.

[119] McManus RM (2014). Respiratory infection promotes T cell infiltration and amyloid-β deposition in APP/PS1 mice. Neurobiol Aging.

[120] Hoffmann M (2020). SARS-CoV-2 cell entry depends on ACE2 and TMPRSS2 and is blocked by a clinically proven protease inhibitor. Cell.

[121] Xu J, Lazartigues E. Expression of ACE2 in Human Neurons Supports the Neuro-Invasive Potential of COVID-19 Virus. Cell Mol Neurobiol. 2020:1–5. 10.1007/s10571-020-00915-1. 10.1007/s10571-020-00915-1 PMC7334623 32623546

[122] Lukiw WJ, Pogue A, Hill JM. SARS-CoV-2 Infectivity and Neurological Targets in the Brain. Cell Mol Neurobiol. 2020:1–8. 10.1007/s10571-020-00947-7. 10.1007/s10571-020-00947-7 PMC7445393 32840758

[123] Netland J (2008). Severe acute respiratory syndrome coronavirus infection causes neuronal death in the absence of encephalitis in mice transgenic for human ACE2. J Virol.

[124] Jiang RD, Liu MQ, Chen Y, Shan C, Zhou YW, Shen XR, Li Q, Zhang L, Zhu Y, Si HR, Wang Q, Min J, Wang X, Zhang W, Li B, Zhang HJ, Baric RS, Zhou P, Yang XL, Shi ZL. Pathogenesis of SARS-CoV-2 in Transgenic Mice Expressing Human Angiotensin-Converting Enzyme 2. Cell. 2020;182(1):50–8.e8. 10.1016/j.cell.2020.05.027. 10.1016/j.cell.2020.05.027 PMC7241398 32516571

[125] Kuba K (2005). A crucial role of angiotensin converting enzyme 2 (ACE2) in SARS coronavirus-induced lung injury. Nat Med.

[126] Glowacka I (2010). Differential downregulation of ACE2 by the spike proteins of severe acute respiratory syndrome coronavirus and human coronavirus NL63. J Virol.

[127] Haga S (2008). Modulation of TNF-α-converting enzyme by the spike protein of SARS-CoV and ACE2 induces TNF-α production and facilitates viral entry. Proc Natl Acad Sci U S A.

[128] Ferreira AJ (2010). Therapeutic implications of the vasoprotective axis of the renin-angiotensin system in cardiovascular diseases. Hypertension.

[129] Paz Ocaranza M (2020). Counter-regulatory renin-angiotensin system in cardiovascular disease. Nat Rev Cardiol.

[130] Arroja MM, Reid E, McCabe C (2016). Therapeutic potential of the renin angiotensin system in ischaemic stroke. Exp Transl Stroke Med.

[131] Chrysant SG (2007). The pathophysiologic role of the brain renin-angiotensin system in stroke protection: clinical implications. J Clin Hypertens (Greenwich).

[132] McCarthy CA, Facey LJ, Widdop RE (2014). The protective arms of the renin-angiontensin system in stroke. Curr Hypertens Rep.

[133] Evans CE (2020). ACE2 activation protects against cognitive decline and reduces amyloid pathology in the Tg2576 mouse model of Alzheimer’s disease. Acta Neuropathol.

[134] Wang XL (2016). Deficiency of angiotensin-converting enzyme 2 causes deterioration of cognitive function. NPJ Aging Mech Dis.

[135] Lanza K (2020). Covid-19: the renin-angiotensin system imbalance hypothesis. Clin Sci (Lond).

[136] Verdecchia P, Cavallini C, Spanevello A, Angeli F. The pivotal link between ACE2 deficiency and SARS-CoV-2 infection. Eur J Intern Med. 2020;76:14–20. 10.1016/j.ejim.2020.04.037. 10.1016/j.ejim.2020.04.037 PMC7167588 32336612

[137] Deshotels MR (2014). Angiotensin II mediates angiotensin converting enzyme type 2 internalization and degradation through an angiotensin II type I receptor-dependent mechanism. Hypertension.

[138] Pasanen L, Launonen H, Siltari A, Korpela R, Vapaatalo H, Salmenkari H, Forsgard RA. Age-related changes in the local intestinal renin-angiotensin system in normotensive and spontaneously hypertensive rats. J Physiol Pharmacol. 2019;70(2). 10.26402/jpp.2019.2.03. 10.26402/jpp.2019.2.03 31356181

[139] Schouten LR (2016). Age-dependent changes in the pulmonary renin-angiotensin system are associated with severity of lung injury in a model of acute lung injury in rats. Crit Care Med.

[140] Xie X (2006). Age- and gender-related difference of ACE2 expression in rat lung. Life Sci.

[141] Yoon HE (2016). Age-associated changes in the vascular renin-angiotensin system in mice. Oxid Med Cell Longev.

[142] Bukowska A (2017). Protective regulation of the ACE2/ACE gene expression by estrogen in human atrial tissue from elderly men. Exp Biol Med (Maywood).

[143] Stelzig KE (2020). Estrogen regulates the expression of SARS-CoV-2 receptor ACE2 in differentiated airway epithelial cells. Am J Physiol Lung Cell Mol Physiol.

[144] Cao Y (2020). Comparative genetic analysis of the novel coronavirus (2019-nCoV/SARS-CoV-2) receptor ACE2 in different populations. Cell Discov.

[145] Li W (2005). Receptor and viral determinants of SARS-coronavirus adaptation to human ACE2. EMBO J.

[146] Grover A, Oberoi M. A systematic review and meta-analysis to evaluate the clinical outcomes in COVID-19 patients on angiotensin-converting enzyme inhibitors or angiotensin receptor blockers. Eur Heart J Cardiovasc Pharmacother. 2020:pvaa064. 10.1093/ehjcvp/pvaa064. 10.1093/ehjcvp/pvaa064 PMC7314072 32542337

[147] Zhang X (2020). ACEI/ARB use and risk of infection or severity or mortality of COVID-19: a systematic review and meta-analysis. Pharmacol Res.

[148] Wang D (2019). Renin-angiotensin-system, a potential pharmacological candidate, in acute respiratory distress syndrome during mechanical ventilation. Pulm Pharmacol Ther.

[149] Wu C, Chen X, Cai Y, Xia J, Zhou X, Xu S, Huang H, Zhang L, Zhou X, Du C, Zhang Y, Song J, Wang S, Chao Y, Yang Z, Xu J, Zhou X, Chen D, Xiong W, Xu L, Zhou F, Jiang J, Bai C, Zheng J, Song Y. Risk Factors Associated With Acute Respiratory Distress Syndrome and Death in Patients With Coronavirus Disease 2019 Pneumonia in Wuhan, China. JAMA Intern Med. 2020;180(7):934–43. 10.1001/jamainternmed.2020.0994. 10.1001/jamainternmed.2020.0994 PMC7070509 32167524

[150] Fan E (2020). COVID-19-associated acute respiratory distress syndrome: is a different approach to management warranted?. Lancet Respir Med.

[151] Doerschug KC (2010). Renin-angiotensin system activation correlates with microvascular dysfunction in a prospective cohort study of clinical sepsis. Crit Care.

[152] Imai Y (2005). Angiotensin-converting enzyme 2 protects from severe acute lung failure. Nature.

[153] Wosten-van Asperen RM (2013). Imbalance between pulmonary angiotensin-converting enzyme and angiotensin-converting enzyme 2 activity in acute respiratory distress syndrome. Pediatr Crit Care Med.

[154] Crowley SD, Rudemiller NP (2017). Immunologic effects of the renin-angiotensin system. J Am Soc Nephrol.

[155] Meng J (2020). Renin-angiotensin system inhibitors improve the clinical outcomes of COVID-19 patients with hypertension. Emerg Microbes Infect.

[156] Rey-Parra GJ (2012). Angiotensin converting enzyme 2 abrogates bleomycin-induced lung injury. J Mol Med (Berl).

[157] Fang Y, Gao F, Liu Z (2019). Angiotensin-converting enzyme 2 attenuates inflammatory response and oxidative stress in hyperoxic lung injury by regulating NF-kappaB and Nrf2 pathways. QJM.

[158] Klein N (2013). Angiotensin-(1-7) protects from experimental acute lung injury. Crit Care Med.

[159] Haddad JJ, Saade NE, Safieh-Garabedian B (2003). Interleukin-10 and the regulation of mitogen-activated protein kinases: are these signalling modules targets for the anti-inflammatory action of this cytokine?. Cell Signal.

[160] Zoufaly A (2020). Human recombinant soluble ACE2 in severe COVID-19. Lancet Respir Med.

[161] Abd El-Aziz, T.M., A. Al-Sabi, and J.D. Stockand, Human recombinant soluble ACE2 (hrsACE2) shows promise for treating severe COVID-19. Signal Transduct Target Ther, 2020. 5(1): p. 258. 10.1038/s41392-020-00374-6 PMC7607365 33144565

[162] Kehoe PG (2017). Angiotensin-III is increased in Alzheimer’s disease in association with amyloid-β and tau pathology. J Alzheimers Dis.

[163] Miners JS (2008). Angiotensin-converting enzyme (ACE) levels and activity in Alzheimer’s disease, and relationship of perivascular ACE-1 to cerebral amyloid angiopathy. Neuropathol Appl Neurobiol.

[164] Miners S (2009). Angiotensin-converting enzyme levels and activity in Alzheimer’s disease: differences in brain and CSF ACE and association with ACE1 genotypes. Am J Transl Res.

[165] Tian M (2012). Central angiotensin II-induced Alzheimer-like tau phosphorylation in normal rat brains. FEBS Lett.

[166] Zhu D (2011). Central angiotensin II stimulation promotes β amyloid production in Sprague Dawley rats. Plos One.

[167] Kehoe PG (2018). The coming of age of the angiotensin hypothesis in Alzheimer’s disease: progress toward disease prevention and treatment?. J Alzheimers Dis.

[168] Chen JL (2017). Angiotensin-(1-7) administration attenuates Alzheimer’s disease-like neuropathology in rats with streptozotocin-induced diabetes via Mas receptor activation. Neuroscience.

[169] Kamel AS (2018). Stimulation of ACE2/ANG(1-7)/Mas axis by diminazene ameliorates Alzheimer’s disease in the d-galactose-ovariectomized rat model: role of PI3K/Akt pathway. Mol Neurobiol.

[170] Griendling KK (1994). Angiotensin II stimulates NADH and NADPH oxidase activity in cultured vascular smooth muscle cells. Circ Res.

[171] Touyz RM, Schiffrin EL (2000). Signal transduction mechanisms mediating the physiological and pathophysiological actions of angiotensin II in vascular smooth muscle cells. Pharmacol Rev.

[172] Naveri L, Stromberg C, Saavedra JM (1994). Angiotensin II AT1 receptor mediated contraction of the perfused rat cerebral artery. Neuroreport.

[173] Matsugi T, Chen Q, Anderson DR (1997). Contractile responses of cultured bovine retinal pericytes to angiotensin II. Arch Ophthalmol.

[174] Kawamura H (2004). Effects of angiotensin II on the pericyte-containing microvasculature of the rat retina. J Physiol.

[175] Fleegal-DeMotta MA, Doghu S, Banks WA (2009). Angiotensin II modulates BBB permeability via activation of the AT(1) receptor in brain endothelial cells. J Cereb Blood Flow Metab.

[176] Zhang M (2010). Angiotensin II induced cerebral microvascular inflammation and increased blood-brain barrier permeability via oxidative stress. Neuroscience.

[177] Carbajo-Lozoya J (2012). Angiotensin II modulates VEGF-driven angiogenesis by opposing effects of type 1 and type 2 receptor stimulation in the microvascular endothelium. Cell Signal.

[178] Kim JH (2009). Blockade of angiotensin II attenuates VEGF-mediated blood-retinal barrier breakdown in diabetic retinopathy. J Cereb Blood Flow Metab.

[179] Tamarat R (2002). Angiotensin II angiogenic effect in vivo involves vascular endothelial growth factor- and inflammation-related pathways. Lab Invest.

[180] Kazama K (2004). Angiotensin II impairs neurovascular coupling in neocortex through NADPH oxidase-derived radicals. Circ Res.

[181] Nishimura Y (1998). The angiotensin AT1 receptor antagonist CV-11974 regulates cerebral blood flow and brain angiotensin AT1 receptor expression. Basic Res Cardiol.

[182] Vraamark T (1995). Angiotensin II receptor antagonist CV-11974 and cerebral blood flow autoregulation. J Hypertens.

[183] Diem AK (2018). A control mechanism for intra-mural peri-arterial drainage via astrocytes: how neuronal activity could improve waste clearance from the brain. Plos One.

[184] Tarasoff-Conway JM (2015). Clearance systems in the brain-implications for Alzheimer disease. Nat Rev Neurol.

[185] Greenberg SM (2020). Cerebral amyloid angiopathy and Alzheimer disease - one peptide, two pathways. Nat Rev Neurol.

[186] Munk AS (2019). PDGF-B is required for development of the glymphatic system. Cell Rep.

[187] Gundersen GA (2014). Evidence that pericytes regulate aquaporin-4 polarization in mouse cortical astrocytes. Brain Struct Funct.

[188] Qin Y (2012). Aquaporin changes during diabetic retinopathy in rats are accelerated by systemic hypertension and are linked to the renin-angiotensin system. Invest Ophthalmol Vis Sci.

[189] Gallagher PE (2006). Distinct roles for ANG II and ANG-(1-7) in the regulation of angiotensin-converting enzyme 2 in rat astrocytes. Am J Physiol Cell Physiol.

[190] Ramanan VK, Saykin AJ (2013). Pathways to neurodegeneration: mechanistic insights from GWAS in Alzheimer’s disease, Parkinson’s disease, and related disorders. Am J Neurodegener Dis.

[191] Tosto G, Reitz C (2013). Genome-wide association studies in Alzheimer’s disease: a review. Curr Neurol Neurosci Rep.

[192] Morgan BP (2018). Complement in the pathogenesis of Alzheimer’s disease. Semin Immunopathol.

[193] Heneka MT (2013). NLRP3 is activated in Alzheimer’s disease and contributes to pathology in APP/PS1 mice. Nature.

[194] Ising C (2019). NLRP3 inflammasome activation drives tau pathology. Nature.

[195] Shagdarsuren E (2005). Complement activation in angiotensin II-induced organ damage. Circ Res.

[196] Zhang C (2014). Complement 5a receptor mediates angiotensin II-induced cardiac inflammation and remodeling. Arterioscler Thromb Vasc Biol.

[197] Zhao M (2018). Angiotensin II stimulates the NLRP3 inflammasome to induce podocyte injury and mitochondrial dysfunction. Kidney Dis (Basel).

[198] Risitano AM (2020). Complement as a target in COVID-19?. Nat Rev Immunol.

[199] Choudhury A, Mukherjee S. In silico studies on the comparative characterization of the interactions of SARS-CoV-2 spike glycoprotein with ACE-2 receptor homologs and human TLRs. J Med Virol. 2020;92(10):2105–13. 10.1002/jmv.25987. 10.1002/jmv.25987 PMC7267663 32383269

[200] De Batista PR (2014). Toll-like receptor 4 upregulation by angiotensin II contributes to hypertension and vascular dysfunction through reactive oxygen species production. Plos One.

[201] Nakashima T (2015). TLR4 is a critical regulator of angiotensin II-induced vascular remodeling: the roles of extracellular SOD and NADPH oxidase. Hypertens Res.

[202] Dange RB (2014). Central blockade of TLR4 improves cardiac function and attenuates myocardial inflammation in angiotensin II-induced hypertension. Cardiovasc Res.

[203] Sheikh BN (2020). Neural metabolic imbalance induced by MOF dysfunction triggers pericyte activation and breakdown of vasculature. Nat Cell Biol.

[204] Toelzer, C., et al., Unexpected free fatty acid binding pocket in the cryo-EM structure of SARS-CoV-2 spike *protein.* bioRxiv 2020. doi: 10.1101/2020.06.18.158584. 10.1126/science.abd3255 PMC8050947 32958580

[205] Shen B (2020). Proteomic and metabolomic characterization of COVID-19 patient sera. Cell.

[206] Snowden SG (2017). Association between fatty acid metabolism in the brain and Alzheimer disease neuropathology and cognitive performance: a nontargeted metabolomic study. Plos Med.

[207] Labandeira-Garcia JL (2017). Brain renin-angiotensin system and microglial polarization: implications for aging and neurodegeneration. Front Aging Neurosci.

[208] Biancardi VC (2016). Cross talk between AT1 receptors and toll-like receptor 4 in microglia contributes to angiotensin II-derived ROS production in the hypothalamic paraventricular nucleus. Am J Physiol Heart Circ Physiol.

[209] Liang B (2015). Angiotensin-(1-7) attenuates angiotensin ii-induced ICAM-1, VCAM-1, and MCP-1 expression via the MAS receptor through suppression of p38 and NF-kappaB pathways in HUVECs. Cell Physiol Biochem.

[210] Rustenhoven J (2017). Brain pericytes as mediators of neuroinflammation. Trends Pharmacol Sci.

[211] Hellner K (2005). Angiotensin-(1-7) enhances LTP in the hippocampus through the G-protein-coupled receptor Mas. Mol Cell Neurosci.

[212] Gironacci MM (2013). Neuromodulatory role of angiotensin-(1-7) in the central nervous system. Clin Sci (Lond).

[213] Bennion DM (2015). Neuroprotective mechanisms of the ACE2-angiotensin-(1-7)-Mas axis in stroke. Curr Hypertens Rep.

[214] Wright JW, Harding JW (2019). Contributions by the brain renin-angiotensin system to memory, cognition, and Alzheimer's disease. J Alzheimers Dis.

[215] Jiang T (2013). ACE2-Ang-(1-7)-Mas axis in brain: a potential target for prevention and treatment of ischemic stroke. Curr Neuropharmacol.

[216] Regenhardt RW, Bennion DM, Sumners C (2014). Cerebroprotective action of angiotensin peptides in stroke. Clin Sci (Lond).

[217] Uekawa K (2016). Intracerebroventricular infusion of angiotensin-(1-7) ameliorates cognitive impairment and memory dysfunction in a mouse model of Alzheimer’s disease. J Alzheimers Dis.

[218] Cao C (2019). Chronic angiotensin 1-7 infusion prevents angiotensin-ii-induced cognitive dysfunction and skeletal muscle injury in a mouse model of Alzheimer’s disease. J Alzheimers Dis.

[219] Roses AD (1996). Apolipoprotein E alleles as risk factors in Alzheimer’s disease. Annu Rev Med.

[220] Tsai MS (1994). Apolipoprotein E: risk factor for Alzheimer disease. Am J Hum Genet.

[221] Montagne A (2020). APOE4 leads to blood-brain barrier dysfunction predicting cognitive decline. Nature.

[222] Love S (2014). Development, appraisal, validation and implementation of a consensus protocol for the assessment of cerebral amyloid angiopathy in post-mortem brain tissue. Am J Neurodegener Dis.

[223] Kuo CL, Pilling LC, Atkins JL, Masoli JAH, Delgado J, Kuchel GA, Melzer D. APOE e4 Genotype Predicts Severe COVID-19 in the UK Biobank Community Cohort. J Gerontol A Biol Sci Med Sci. 202015;75(11):2231–2. 10.1093/gerona/glaa131. 10.1093/gerona/glaa131 PMC7314139 32451547

[224] Yamazaki Y (2020). ApoE (Apolipoprotein E) in brain pericytes regulates endothelial function in an isoform-dependent manner by modulating basement membrane components. Arterioscler Thromb Vasc Biol.

[225] Thambisetty M (2010). APOE ϵ4 genotype and longitudinal changes in cerebral blood flow in normal aging. Arch Neurol.

[226] Koizumi K (2018). Apoϵ4 disrupts neurovascular regulation and undermines white matter integrity and cognitive function. Nat Commun.

[227] Kloske CM, Wilcock DM (2020). The important interface between apolipoprotein E and neuroinflammation in Alzheimer’s disease. Front Immunol.

[228] Patel VB (2014). Angiotensin II induced proteolytic cleavage of myocardial ACE2 is mediated by TACE/ADAM-17: a positive feedback mechanism in the RAS. J Mol Cell Cardiol.

[229] Gooz M (2010). ADAM-17: the enzyme that does it all. Crit Rev Biochem Mol Biol.

[230] Cui S (2020). Prevalence of venous thromboembolism in patients with severe novel coronavirus pneumonia. J Thromb Haemost.

[231] Klok FA (2020). Incidence of thrombotic complications in critically ill ICU patients with COVID-19. Thromb Res.

[232] Martin K (2016). Use of the direct oral anticoagulants in obese patients: guidance from the SSC of the ISTH. J Thromb Haemost.

[233] Thachil J (2020). ISTH interim guidance on recognition and management of coagulopathy in COVID-19. J Thromb Haemost.

[234] Aryal MR (2020). Venous thromboembolism in COVID-19: towards an ideal approach to thromboprophylaxis, screening, and treatment. Curr Cardiol Rep.

[235] Cao X (2020). COVID-19: immunopathology and its implications for therapy. Nat Rev Immunol.

[236] Hegerova L (2020). Use of convalescent plasma in hospitalized patients with COVID-19: case series. Blood.

[237] Gucyetmez B (2020). Therapeutic plasma exchange in patients with COVID-19 pneumonia in intensive care unit: a retrospective study. Crit Care.

[238] Morath C (2020). Plasma exchange in critically ill COVID-19 patients. Crit Care.

[239] Fernandez-Ruiz M, et al. Tocilizumab for the treatment of adult patients with severe COVID-19 pneumonia: a single-center cohort study. J Med Virol. 2020;. 10.1002/jmv.26308 PMC7404673 32672860

[240] Montesarchio V, Parrela R, Iommelli C, Bianco A, Manzillo E, Fraganza F, Palumbo C, Rea G, Murino P, De Rosa R, Atripaldi L, D'Abbraccio M, Curvietto M, Mallardo D, Celentano E, Grimaldi AM, Palla M, Trojaniello C, Vitale MG, Million-Weaver SL, Ascierto PA. Outcomes and biomarker analyses among patients with COVID-19 treated with interleukin 6 (IL-6) receptor antagonist sarilumab at a single institution in Italy. J Immunother Cancer. 2020;8(2):e001089. 10.1136/jitc-2020-001089. 10.1136/jitc-2020-001089 PMC7418768 32784217

[241] Leng Z (2020). Transplantation of ACE2(−) mesenchymal stem cells improves the outcome of patients with COVID-19 pneumonia. Aging Dis.

[242] Cheng Z (2018). Mesenchymal stem cells attenuate blood-brain barrier leakage after cerebral ischemia in mice. J Neuroinflammation.

[243] Chao YX, He BP, Tay SS (2009). Mesenchymal stem cell transplantation attenuates blood brain barrier damage and neuroinflammation and protects dopaminergic neurons against MPTP toxicity in the substantia nigra in a model of Parkinson’s disease. J Neuroimmunol.

[244] Tang G (2014). Mesenchymal stem cells maintain blood-brain barrier integrity by inhibiting aquaporin-4 upregulation after cerebral ischemia. Stem Cells.

[245] Russell CD, Millar JE, Baillie JK (2020). Clinical evidence does not support corticosteroid treatment for 2019-nCoV lung injury. Lancet.

[246] Horby, P., et al., Effect of dexamethasone in hospitalized patients with COVID-19: preliminary report. medRxiv, 2020. doi: 10.1101/2020.06.22.20137273**.**.

[247] Artigas A (2017). Inhalation therapies in acute respiratory distress syndrome. Ann Transl Med.

[248] Ortiz-Diaz E (2013). Emerging pharmacological therapies for prevention and early treatment of acute lung injury. Semin Respir Crit Care Med.

[249] Yamaya M (2020). Inhibitory effects of glycopyrronium, formoterol, and budesonide on coronavirus HCoV-229E replication and cytokine production by primary cultures of human nasal and tracheal epithelial cells. Respir Investig.

[250] Jeon S, Ko M, Lee J, Choi I, Byun SY, Park S, Shum D, Kim S. Identification of antiviral drug candidates against SARS-CoV-2 from FDA-approved drugs. Antimicrob Agents Chemother. 2020;64(7):e00819–20. 10.1128/AAC.00819-20. 10.1128/AAC.00819-20 PMC7318052 32366720

[251] Hadjadj J (2020). Impaired type I interferon activity and inflammatory responses in severe COVID-19 patients. Science.

[252] Felgenhauer U, Schoen A, Gad HH, Hartmann R, Schaubmar AR, Failing K, Drosten C, Weber F. Inhibition of SARS-CoV-2 by type I and type III interferons. J Biol Chem. 2020;295(41):13958–64. 10.1074/jbc.AC120.013788. 10.1074/jbc.AC120.013788 PMC7549028 32587093

[253] Hung IF (2020). Triple combination of interferon β-1b, lopinavir-ritonavir, and ribavirin in the treatment of patients admitted to hospital with COVID-19: an open-label, randomised, phase 2 trial. Lancet.

[254] Fang L, Karakiulakis G, Roth M (2020). Are patients with hypertension and diabetes mellitus at increased risk for COVID-19 infection?. Lancet Respir Med.

[255] Flacco ME, Acuti Martellucci C, Bravi F, Parruti G, Cappadona R, Mascitelli A, Manfredini R, Mantovani LG, Manzoli L. Treatment with ACE inhibitors or ARBs and risk of severe/lethal COVID-19: a meta-analysis. Heart. 2020;106(19):1519–24. 10.1136/heartjnl-2020-317336. 10.1136/heartjnl-2020-317336 PMC7371482 32611676

[256] Rizk JG, Kalantar-Zadeh K, Mehra MR, Lavie CJ, Rizk Y, Forthal DN. Pharmaco-Immunomodulatory Therapy in COVID-19. Drugs. 2020;80(13):1267–92. 10.1007/s40265-020-01367-z. 10.1007/s40265-020-01367-z PMC7372203 32696108

[257] Alijotas-Reig J (2020). Immunomodulatory therapy for the management of severe COVID-19. Beyond the anti-viral therapy: a comprehensive review. Autoimmun Rev.

[258] Chibber P, Haq SA, Ahmed I, Andrabi NI, Singh G. Advances in the possible treatment of COVID-19: A review. Eur J Pharmacol. 2020;883:173372. 10.1016/j.ejphar.2020.173372. 10.1016/j.ejphar.2020.173372 PMC7366101 32682787

[259] Bianchetti A (2020). Clinical presentation of COVID19 in dementia patients. J Nutr Health Aging.

[260] Hwang JM (2020). Neurological diseases as mortality predictive factors for patients with COVID-19: a retrospective cohort study. Neurol Sci.

